# An integrated analysis of mRNAs, lncRNAs, and miRNAs based on weighted gene co-expression network analysis involved in bovine endometritis

**DOI:** 10.1038/s41598-021-97319-y

**Published:** 2021-09-10

**Authors:** Negin Sheybani, Mohammad Reza Bakhtiarizadeh, Abdolreza Salehi

**Affiliations:** Department of Animal and Poultry Science, College of Aburaihan, University of Tehran, Tehran, Iran

**Keywords:** Non-coding RNAs, Transcriptomics

## Abstract

In dairy cattle, endometritis is a severe infectious disease that occurs following parturition. It is clear that genetic factors are involved in the etiology of endometritis, however, the molecular pathogenesis of endometritis is not entirely understood. In this study, a system biology approach was used to better understand the molecular mechanisms underlying the development of endometritis. Forty transcriptomic datasets comprising of 20 RNA-Seq (GSE66825) and 20 miRNA-Seq (GSE66826) were obtained from the GEO database. Next, the co-expressed modules were constructed based on RNA-Seq (Rb-modules) and miRNA-Seq (mb-modules) data, separately, using a weighted gene co-expression network analysis (WGCNA) approach. Preservation analysis was used to find the non-preserved Rb-modules in endometritis samples. Afterward, the non-preserved Rb-modules were assigned to the mb-modules to construct the integrated regulatory networks. Just highly connected genes (hubs) in the networks were considered and functional enrichment analysis was used to identify the biological pathways associated with the development of the disease. Furthermore, additional bioinformatic analysis including protein–protein interactions network and miRNA target prediction were applied to enhance the reliability of the results. Thirty-five Rb-modules and 10 mb-modules were identified and 19 and 10 modules were non-preserved, respectively, which were enriched in biological pathways related to endometritis like inflammation and ciliogenesis. Two non-preserved Rb-modules were significantly assigned to three mb-modules and three and two important sub-networks in the Rb-modules were identified, respectively, including important mRNAs, lncRNAs and miRNAs genes like *IRAK1*, *CASP3*, *CCDC40*, *CCDC39*, *ZMYND10*, *FOXJ1*, *TLR4*, *IL10*, *STAT3*, *FN1*, *AKT1*, *CD68*, *ENSBTAG00000049936*, *ENSBTAG00000050527*, *ENSBTAG00000051242*, *ENSBTAG00000049287*, *bta-miR-449*, *bta-miR-484*, *bta-miR-149*, *bta-miR-30b* and *bta-miR-423*. The potential roles of these genes have been previously demonstrated in endometritis or related pathways, which reinforced putative functions of the suggested integrated regulatory networks in the endometritis pathogenesis. These findings may help further elucidate the underlying mechanisms of bovine endometritis.

## Introduction

One of the main goals of reproductive management in dairy cattle is leading cows to be pregnant at a normal calving interval and in an effective manner^1,2^. Although several reasons currently exist for reproductive inefficiency in dairy cows, postpartum uterine disease is considered as one of the most important causes in this regard^3^. Infection of the postpartum uterus with bacteria, namely endometritis, affects half of all dairy cattle and consequently causes infertility or subfertility by interrupting both uterine and ovarian functions^4^. Postpartum endometritis (during 21 days or more after calving) is commonly observed among high-producing dairy cows, which could adversely affect their reproductive performance by increasing services per conception and calving interval, decreasing the risk of pregnancy and the rate of conception, and reducing milk yield that consequently result in economic loss^5^. In this context, the annual economic losses from cattle uterine disorders were estimated to be $1.4 billion and $650 million in Europe and the United States, respectively^4^. Undoubtedly, the development of endometritis stems from some complex interactions between bacteria and host factors, and genetic factors play important roles in the development of the disease as well^6^. However, the molecular genetic mechanisms by which endometritis causes infertility or subfertility are not fully clear yet.

Several genes, transcription factors (TFs), biological processes and signaling pathways have been reported in the previous studies to be involved in the development of endometritis. Investigation of gene expression profile using real time-PCR (RT-PCR) showed the higher expression of *CXCL5*, *IL1B*, *IL6*, *IL8*, *PTGS2* and *TNF* in bovine with an inflamed endometrium in comparison to cows with a healthy endometrium^7^. A higher expression of *PTGS2* gene, which is involved in the inflammatory response, was detected in the repeat breeder cows with subclinical endometritis compared to healthy repeat breeder cows (using RT-PCR)^8^. Nuclear factor *(NF)-κB*, as a TF, has been also reported as a major regulator of pro‐inflammatory genes involving in the pathogenesis of endometritis^9,10^. It was found that cows with subclinical endometritis at 45–55 days postpartum had higher expression of immune factors *C3*, *C2*, *LTF*, *PF4* and *TRAPPC13* (using microarray) in comparison to healthy cows at the same time points^11^. Transcriptome profile analysis of Holstein Frisian cows (with and without clinical endometritis) at 42–60 days postpartum revealed that 92 genes including *PTHLH*, *INHBA*, *DAPL1* and *SERPINA1* were significantly upregulated (using quantitative RT-PCR) in clinical endometritis cows compared to the healthy animal group^12^.

The literature review showed that most of the previously performed studies have focused on the gene expression analysis, which only considers the differentially expressed genes and does not consider the interactions among them. Therefore, to fill this gap, the co-expressed gene regulatory network constructed based on the gene expression data, in order to better explain the complex etiology of biological processes or some diseases like endometritis^13^. In this regard, the weighted gene co-expression network analysis (WGCNA) is a popular and effective gene co-expression network-based approach, which is performed based on the “guilt-by-association” method^14^. WGCNA measures the connectivity among the genes based on the gene expression patterns and clusters of the highly correlated genes into modules by considering their expression similarities^15,16^. Moreover, WGCNA could assess the preservation of the modules to identify those modules with topological differences between those situations (such as normal vs diseases)^16^. It was reported that some modules that lost their preservation in other situations may be involved in the development of that situations^17^, so they can be considered as potential candidates for further studies^18^. In our previous study for the first time, WGCNA was applied to better understand the molecular mechanisms of the endometriosis. To do this, publicly available microarray data from women with minimal/mild endometriosis, mild/severe endometriosis and without endometriosis were used. In total, 16 functional modules were found in the normal samples as reference, of which nine modules were non-preserved in the two other stages. Several important hub genes were also suggested for further investigations including *NF-kB*, *IPO9*, *PTPMT1* and *PTGDS*^19^.

More recent evidence showed that non-coding RNAs (ncRNAs), especially microRNAs (miRNAs)^20–22^ and long ncRNAs (lncRNAs)^23,24^, play important roles in different aspects of the biological processes, including innate and adaptive immunity and inflammation^25^. Furthermore, they can be used as biomarkers or therapeutic targets in different diseases like endometritis^26^. Moreover, lncRNAs are transcripts without any known protein coding potential greater than 200 nt in length^27^. Many biological processes have been found to have associations with lncRNAs at three levels of transcriptional, post transcriptional, and translational^28^. As well, the regulatory role of lncRNA *Mirt2* has been demonstrated in both inflammation and macrophage polarization^29^. Many other lncRNAs such as *THRIL*, *PACER*, *Lethe*, *NKILA*, and *lnc13*, have been also identified as crucial regulators in inflammation and immune system^30^. MiRNAs, as another class of ncRNAs, are ∼ 22-nt long, which could post-transcriptionally regulate the expression of targeted mRNAs^31^. Disrupting some miRNAs regulatory mechanisms or losing some of them can lead to disorders in immune system^32^. Deregulation of *miR-203* was also identified as a potential biomarker of psoriasis development (as one of the most prevalent chronic inflammatory skin diseases among adults)^33^. Many other miRNAs such as *miR-223*, *miR-146a*, *miR-146b*, *miR-125a*, *miR-125b*, *miR‐148a*, and *miR-21* have been found to be associated with inflammatory diseases or immune responses^22,34^. Therefore, gene co-expression network construction in the presence of both mRNAs and ncRNAs may provide more biological insights compared to when performing separate analyses to study complex diseases. This type of the integrated regulatory network can help in exploring the roles of ncRNAs in endometritis onset and development.

In the present study, we hypothesized that integrating mRNAs and ncRNAs expression profiles (as miRNAs and lncRNAs) used to construct an integrated co-expression network would provide additional insights into the endometritis-associated biological pathways. Therefore, in the present study, WGCNA was applied to identify the co-expression RNA-Seq-based modules (Rb-modules) (including mRNAs and lncRNAs) and miRNA-Seq-based modules (mb-modules) in healthy samples. Afterward, to find the non-preserved Rb-modules in the endometritis samples, preservation analysis was used. Next, the mb-modules were assigned to the non-preserved Rb-modules and then an integrated co-expression network was constructed. As well, protein–protein interaction network, functional enrichment analysis, miRNA target prediction, and hub gene detection approaches were applied to help in identifying the most important candidate genes, which may consequently affect the development of endometritis. Our findings may further aid research studies to provide novel insights into the pathogenesis of endometritis and/or identifying therapeutic targets in this regard.

## Material and methods

### Dataset

Raw RNA-Seq and miRNA-Seq data were obtained from the Gene Expression Omnibus (GEO) database at the National Center for Biotechnology Information (NCBI) under the accession number GSE66827. The data contained endometrial samples related to nine healthy and six endometritis cows at 7 and 21 days post-partum^6^. In the early postpartum period, healthy cows can be differentiated from cows at risk of developing uterine disease through change in immune profile. Therefore, all cows at two time points postpartum during uterine involution were examined for the inflammation extension and categorized as either healthy or having subclinical endometritis. The Illumina^®^ HiSeq™ 2000 was used for sequencing. Finally, 30 RNA-Seq libraries containing 49 bp reads and 20 miRNA-Seq libraries containing 51 bp reads were generated. More details of preparing data can be found in the original paper^6^.

### RNA-Seq and miRNA-Seq data analysis

Here, to construct an integrated regulatory network, only cows with available RNA-Seq and miRNA-Seq data were considered. Hence, 20 RNA-Seq (GSE66825) and 20 miRNA-Seq (GSE66826) samples were retained for further analysis. The quality of raw RNA-Seq and miRNA-Seq reads were checked by FastQC (version 0.11.5)^35^. Low quality bases/reads and adaptor sequences for both RNA-Seq and miRNA-Seq reads were removed by Trimmomatic (version 0.32)^36^. The trimming criteria for RNA-Seq data was TRAILING: 20, MAXINFO: 40: 0.9, MINLEN: 40 and for miRNA-Seq data was TRAILING: 20, MAXINFO: 18:0.9, MINLEN: 18. Then, FastQC was used again for monitoring the quality of the reads after trimming.

Clean reads from RNA-Seq data were aligned to bovine reference genome (version ARS-UCD1.2) using Hisat2 (version 2.1.0)^37^. Then, Python script HTSeq-count (version 2.7.3) was used to count the RNA-Seq read numbers mapped to annotated genes using the Ensembl bovine GTF file (release 98)^38^. The generated gene expression matrix included mRNAs and lncRNAs. To identify transcription factors (TFs) in the expression matrix, AnimalTFDB3.0 database was used^39^. For miRNA-Seq data, non-miRNA reads were filtered out using Unitas (version 1.7.0)^40^. Bowtie software (version 1.3.0) was then used to align miRNA-based reads to bovine pre-mature miRNA sequences downloaded from miRBase database (version 22) with mismatch ≤ 2^41^. Finally, SAMtools (version 1.9) was used to quantify the expression of each miRNA and generate miRNA expression matrix^42^.

Taken together, WGCNA is expanded as an unsupervised approach to analyze microarray data sets^43^, both expression matrix that were included raw count data were normalized to log counts per million (CPM) based on voom approach in limma package of R software (version 3.42.0)^44^. On the other hand, reliability of the genes with low expression levels is not high enough to be used in co-expression analysis^45^. Hence, for tackling this issue, genes in both expression matrices were assessed and the genes with expression levels ≥ 1 CPM in at least four samples as well as standard deviation higher than 0.25 were kept for further analysis.

### Weighted co-expression network analysis

Rb- and mb-modules were constructed separately by WGCNA R package (version 1.68)^16^. To ensure whether network construction was reliable, the outlier samples were excluded. For this purpose, adjacency matrices of both expression matrices matrix were calculated independently and sample network connectivity according to the distances was standardized. Outlier samples were defined as samples with a standardized connectivity less than − 2.5. Then to determine whether the sample data were complete as well as exclude the unqualified genes, goodSamplesGenes function in WGCNA package was applied.

To construct Rb-modules, an appropriate soft-thresholding power was calculated to be confident that the constructed network follows the scale-free topology. The scale-free network contains several nodes with few interactions and few nodes with high interactions, which is called hub nodes^46^. The approximate scale-free topology in healthy samples was achieved at a soft threshold of β = 13. Afterward, Rb-modules were constructed using blockwiseModules function in the WGCNA package, with the following major parameters: power = 13, corType = “bicor”, networkType = “signed”, TOMType = “signed”, maxBlockSize = 17,000, minModuleSize = 30, reassignThreshold = 0, mergeCutHeight = 0.25. Also, β = 22 met the soft-threshold parameters for signed miRNA co-expression network construction and the curve reached R^2^ = 0.80. For mb-module detection, blockwiseModules function in the WGCNA package was used with the following major parameters: power = 22, corType = “bicor”, networkType = “signed”, TOMType = “signed”, maxBlockSize = 17,000, minModuleSize = 10, reassignThreshold = 0, mergeCutHeight = 0.25. Because of the smaller size of the miRNA transcriptome in comparison to the mRNA-lncRNA transcriptome, minimum module size for the miRNAs was set as 10^16,47^. It is worth to note that, to identify the modules in both datasets, first, based on the soft-thresholding power, weighted adjacency matrices was constructed and was transformed into the topological matrix (TOM), which describe the association strength between the genes. Then, TOM based adjacency matrices were clustered using average linkage hierarchical clustering analysis through a dynamic hybrid tree cutting algorithm and the highly similar modules were further merged, based on the correlations between the module eigengenes.

### Preservation analysis

Using modulePreservation function in WGCNA package, it was investigated whether the connectivity patterns of the genes in the detected Rb-modules in healthy samples are preserved in the infected samples. Two composite module preservation statics including Zsummary and medianRank were applied to evaluate the preservation, based on connectivity and density of genes present in a module. Since Zsummary depends on the module size, especially when modules with different sizes have to be compared, medianRank, as an index that is less sensitive to the module size, can be combined with Zsummary to accurately test the preservation level. The higher value of Zsummary and the lower value of medianRank indicate strong preservation in the infected samples^48^. The Zsummary < 5 and MedianRank > 8 were considered as criteria for non-preserved modules. A 5 < Zsummary < 10 and MedianRank > 8 indicated evidence of semi-preserved modules. If Zsummary > 10 and MedianRank < 8, it was concluded that there was evidence of module preservation. Permutation method (N = 200 permutations) was used to evaluate statistical significance of both module preservation statics.

### Detection of hub genes

It is well known that all the genes present in a module are not important to be linked with the subject of interest (here endometritis). Hence, some genes are more interesting than others in topological and functional perspective, called hub genes. In this context, the scale-free property of the biological networks indicates to this point that a small number of highly connected genes hold the network together. The WGCNA functions moduleEigengenes and signedKME were used to identify the hub mRNAs, lncRNAs and miRNAs in the relevant modules. In both of the constructed modules, expression profile of each module was summarized by module eigengene (MEs). MEs refers to the primary component in the principal component analysis of the genes in a module^49^. To quantify the relationship between a gene and a given module, kME or module membership was determined as the correlation between the expression profile of a gene and the ME of a module^16^. kME describes the degree of internal connectivity of the module or the degree of association between a gene in a particular module and other genes in the module. Hub genes were determined as the genes with kME ≥ 0.7^16^.

### Functional enrichment analysis

To elucidate the putative biological functions of the selected genes (genes of each module, hub- or hub-hub genes), GO terms (biological process) and Kyoto Encyclopedia of Genes and Genomes (KEGG) pathway enrichment analysis were performed using the Enrichr online analysis tool (https://maayanlab.cloud/Enrichr/). Only significant terms with adjusted p-values < 0.05 were considered.

### Assign the mb-modules to the Rb-modules and target prediction

To assign the mb-modules to Rb-modules, spearman correlations were calculated between the MEs of Rb-modules and mb-modules. Negative correlations suggest that mb-modules might inversely regulate Rb-modules. Hence, mb-modules with a significant (adjusted p-value < 0.20) negative correlation larger than 0.80 were defined as regulators of the Rb-module of interest.

RNAhybrid (version 2.2)^50^, miRanda (version 3.3a)^51^ and RNA22 (version 2.0)^52^ were applied to predict the targets of miRNAs in the mb-modules that were negatively correlated with a Rb-module. In this respect, bovine 3′ UTR sequences (mRNAs and lncRNAs genes) were retrieved from Ensemble database (https://www.ensembl.org/). Also, bovine mature miRNA sequences were obtained from miRBase database (version 22). Minimum free energy threshold was set as − 15 and other parameters were set as default for all used software. To avoid false positive results, the predicted targets for each miRNA were considered as potential targets, if were detected by at least two out of the three applied software.

### Detection of hub-hub genes in Rb-modules

In order to identify the most important hub genes (hub-hub genes) in the Rb-modules with a significant assigned mb-module, the following steps were performed:

1. Protein–protein interactions (PPIs) among the mRNA hub genes of each module were obtained using Search Tool for the Retrieval of Interacting Genes (STRING) database^53^.
2. All the identified interactions related to hub mRNAs (including STRING-PPIs and WGCNA-calculated co-expressed mRNAs) and hub lncRNAs (WGCNA-calculated co-expressed lncRNAs) of each module were inputted to Cytoscape software (version 3.7.2) (https://cytoscape.org/) and were evaluated by cytoHubba application (version 0.1)^54^. CytoHubba suggests 12 topological analysis methods including maximum click centrality (MCC), density of maximum neighborhood component (DMNC), maximum neighborhood component (MNC), degree method, edge percolated component (EPC), bottleneck, EcCentricity, closeness, radiality, betweenness, stress and clustering coefficient to screen the highly connected genes in a given network^54^. All the methods were applied to detect the hub-hub genes in each module.
3. Once all the results from the 12 methods were obtained, the RankAggreg package (version 0.6.5) in R software^55^ was used to connect the results of all methods with each other and generate a consensus ranking. RankAggreg package ordered lists based on the Cross-Entropy Monte Carlo algorithm and the Genetic Algorithm. For increasing the accuracy of the result, both methods were applied and the genes that were identified as highly connected genes in both methods were reported as hub-hub genes.

### Integrated regulatory network

Cytoscape (version 3.7.2) was used to combine all the identified interactions among the genes and visualize the final integrated regulatory network. For each module, the obtained interactions from STRING-PPIs, WGCNA-calculated co-expressed mRNAs, WGCNA-calculated co-expressed lncRNAs and WGCNA-calculated co-expressed miRNAs (if an mb-module was assigned to the Rb-module) were used to establish the integrated regulatory network.

## Results

### RNA-Seq and miRNA-Seq data analysis

**Figure 1:**
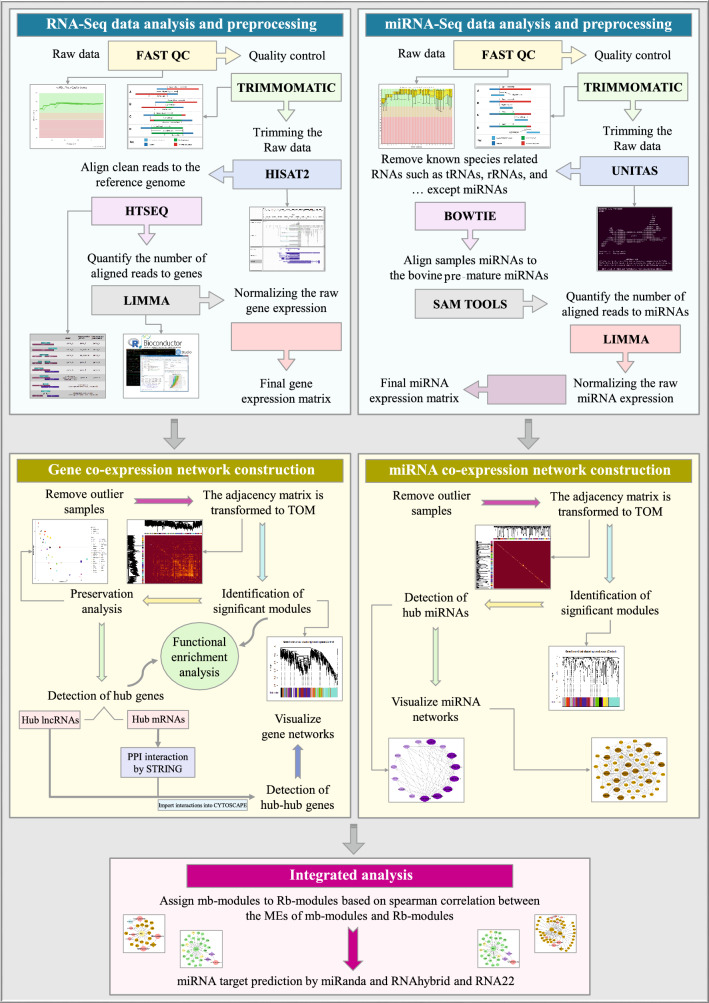
The used pipeline for constructing the integrated regulatory network.

An overview of the used pipeline is provided in Fig. 1. In total, 785,810,232 RNA-Seq and 350,319,753 miRNA-Seq reads were processed in this study. After checking the quality and trimming the reads, 758,465,299 RNA-Seq and 334,769,250 miRNA-Seq reads were remained. On average, 91.58% of RNA-Seq and 79.95% of miRNA-Seq reads were aligned to the reference genome and bovine pre-mature miRNAs, respectively. The results of sequencing and mapping are presented in Supplementary File S1. Gene expression analysis and screening out the less-expressed ones led to generate a normalized gene expression matrix containing 11,744 genes (10,940 mRNAs, 775 TFs and 190 lncRNAs) and a normalized miRNA expression matrix containing 420 miRNAs.

### Detection of co-expressed genes

**Figure 2:**
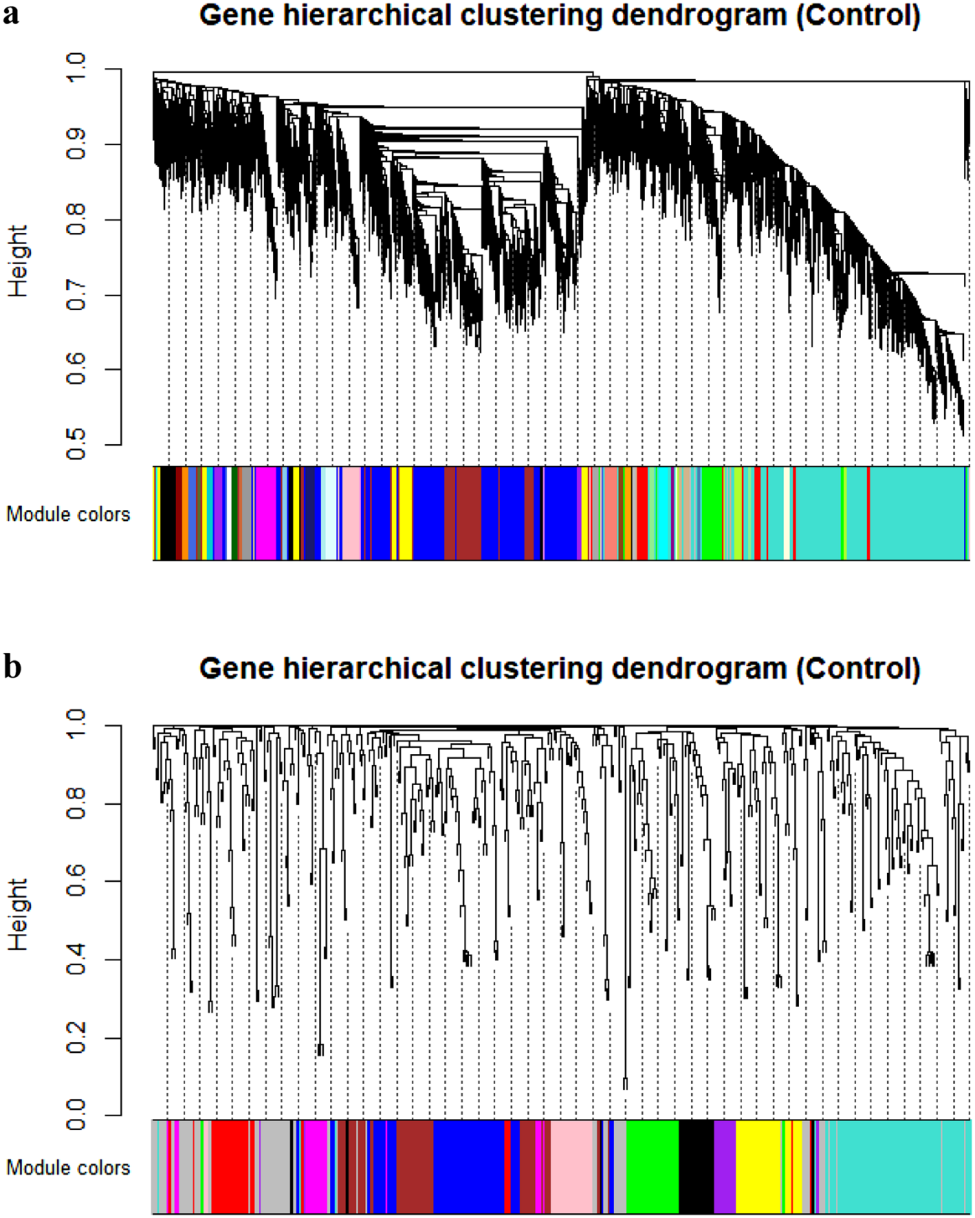
Clustering dendrogram of mRNAs and lncRNAs (**a**) and miRNAs (**b**). In WGCNA genes are clustered into modules based on their co-expression. Hierarchical clustering is a widely used method for detecting clusters. The co-expressed genes have a small degree of dissimilarity. The dissimilarity measure (1-TOM) can be used as input in hierarchical clustering. Following that, modules were defined as branches of a cluster tree and each module was labeled by a unique color using the static tree cutting method.

**Figure 3:**
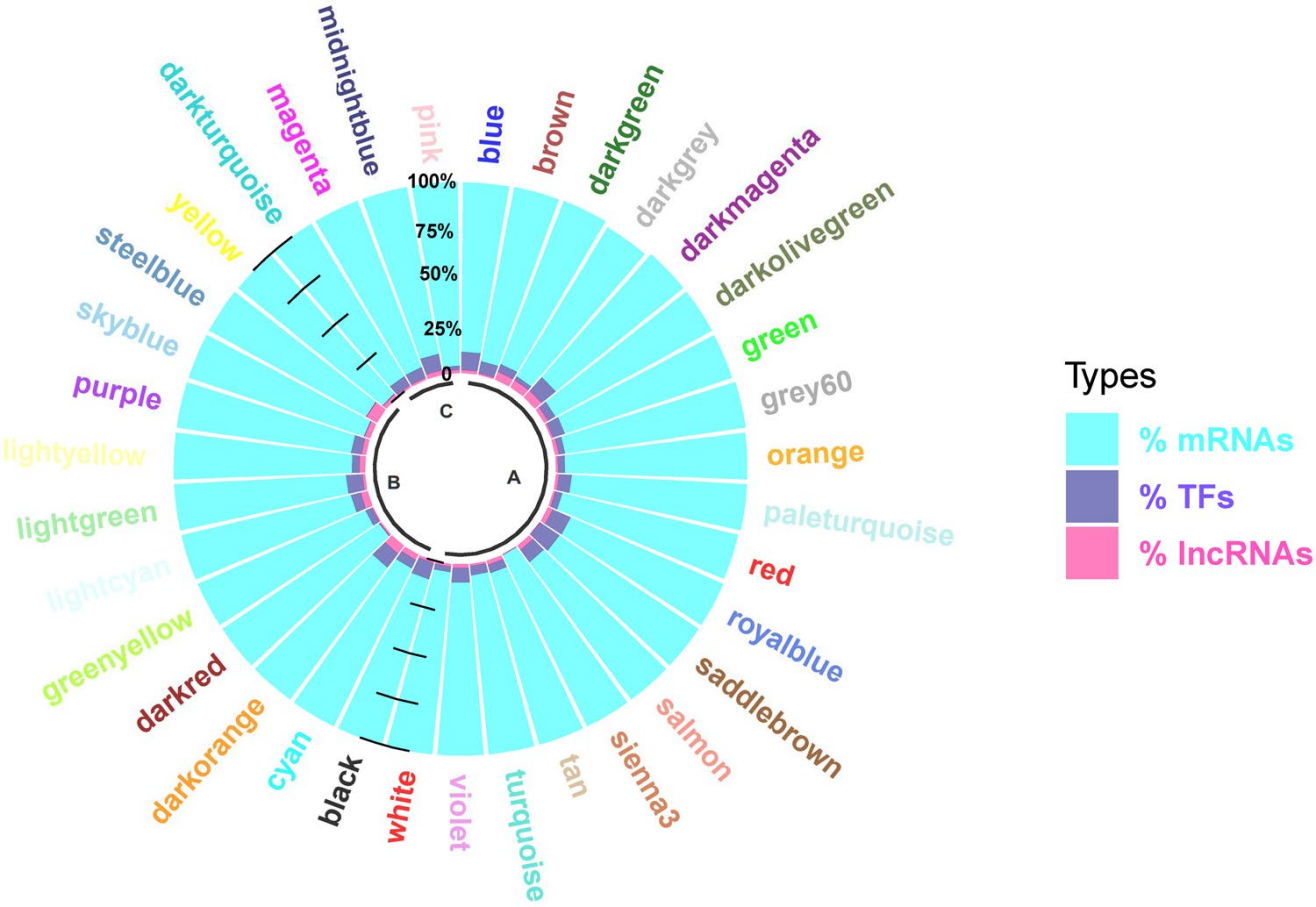
Percentage of mRNAs, lncRNAs and TFs in each Rb-module. Rb-modules were identified across 20 RNA-Seq datasets. A, B and C region indicate non-preserved, semi-preserved and preserved Rb-modules, respectively. As it can be seen, the percentage of mRNAs in all modules is considerably higher than percentage of TFs and lncRNAs. Despite the important regulatory role of TFs and lncRNAs, almost less than 15% of genes in each module is devoted to them.

In this study, a network-based approach was applied to establish a better understanding on the molecular mechanisms of endometritis. No outlier samples were observed in the given datasets. After the determination of the appropriate soft threshold power beta (Supplementary File S2), 35 Rb-modules and 10 mb-modules were detected by hierarchical clustering and dynamic branch cutting, which were labelled by different colors based on the WGCNA approach (Fig. 2). The average number of genes per module in the Rb- and mb-modules were obtained as 333 and 35, respectively. Accordingly, the largest Rb-module was turquoise module (containing 3188 genes, including 152 TFs, 36 lncRNAs, and 2973 mRNAs), while the smallest module was sienna3 (containing 36 mRNAs). The greatest and smallest numbers of lncRNAs belonged to blue with 40 lncRNAs, saddlebrown, and sienna3 with no lncRNAs. Using the AnimalTFDB database, a total of 682 TFs were identified in the Rb-modules (Supplementary File S3), in which blue module with 224 TFs and sienna3, darkred, skyblue, steelblue, and yellow module with no TFs had the greatest and smallest numbers of TFs (Fig. 3). Among the mb-modules, turquoise was found as the largest one and the purple was found as the smallest one with 74 and 15 miRNAs, respectively. Additionally, 102 and 69 genes were reported as grey modules in Rb- and mb-modules, respectively, which contained some genes that were not assigned to any module. Details of all the genes of each module are represented in Supplementary File S4.

### Preservation analysis

**Figure 4:**
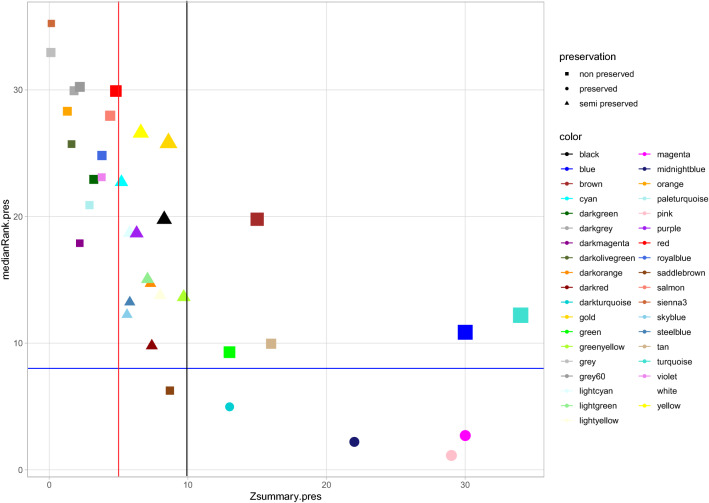
The medianRank (y axis) and Zsummary (x axis) statistics of the module preservation across 20 RNA-Seq datasets. Non-preserved, semi-preserved and preserved modules are indicated by square, circle and triangle points respectively in Rb-modules. The red and black vertical lines indicate the thresholds Zsummary = 8 and Zsummary = 10 respectively. The blue horizontal line indicates a threshold MedianRank = 8. The modules with Zsummary < 5 and MedianRank > 8 were considered as non-preserved, 5 < Zsummary < 10 and MedianRank > 8 semi-preserved and Zsummary > 10 and MedianRank < 8 preserved.

The preservation analysis of Rb-modules was performed to dissect the connectivity patterns between the two healthy and endometritis conditions. Network properties of non- and semi-preserved modules were altered under endometritis compared to healthy conditions, so they may be related to the development of endometritis. Of 35 Rb-modules, 19, 12 and four modules were detected as non-preserved, semi-preserved and preserved modules, respectively (Fig. 4, Supplementary File S5).

Hub genes show higher connectivity inside the module and are probably more informative^56^. In total, 635 (including 582 mRNAs, 34 TFs and 10 lncRNAs), 5310 (including 4831 mRNAs, 366 TFs and 71 lncRNAs) and 1437 (including 1332 mRNAs, 60 TFs and 33 lncRNAs) hub genes were identified in preserved, non- and semi-preserved Rb-modules, respectively (Supplementary File S6). The highest number of hub genes was found in turquoise (1910 genes including 1,788 mRNAs, 89 TFs and 22 lncRNAs), blue (1301 genes including 1125 mRNAs, 147 TFs 19 lncRNAs) and brown (692 genes including 632 mRNAs, 49 TFs and 6 lncRNAs) Rb-modules, respectively. The total number of hub miRNAs was 212 (Supplementary File S7). Blue, turquoise and brown mb-modules showed the highest number of hub miRNAs including 37, 36 and 25, respectively. In contrast, sienna3 non-preserved Rb-module and purple mb-module had the lowest number of hubs including 30 and 13 genes, respectively.

### Functional enrichment analysis

**Figure 5:**
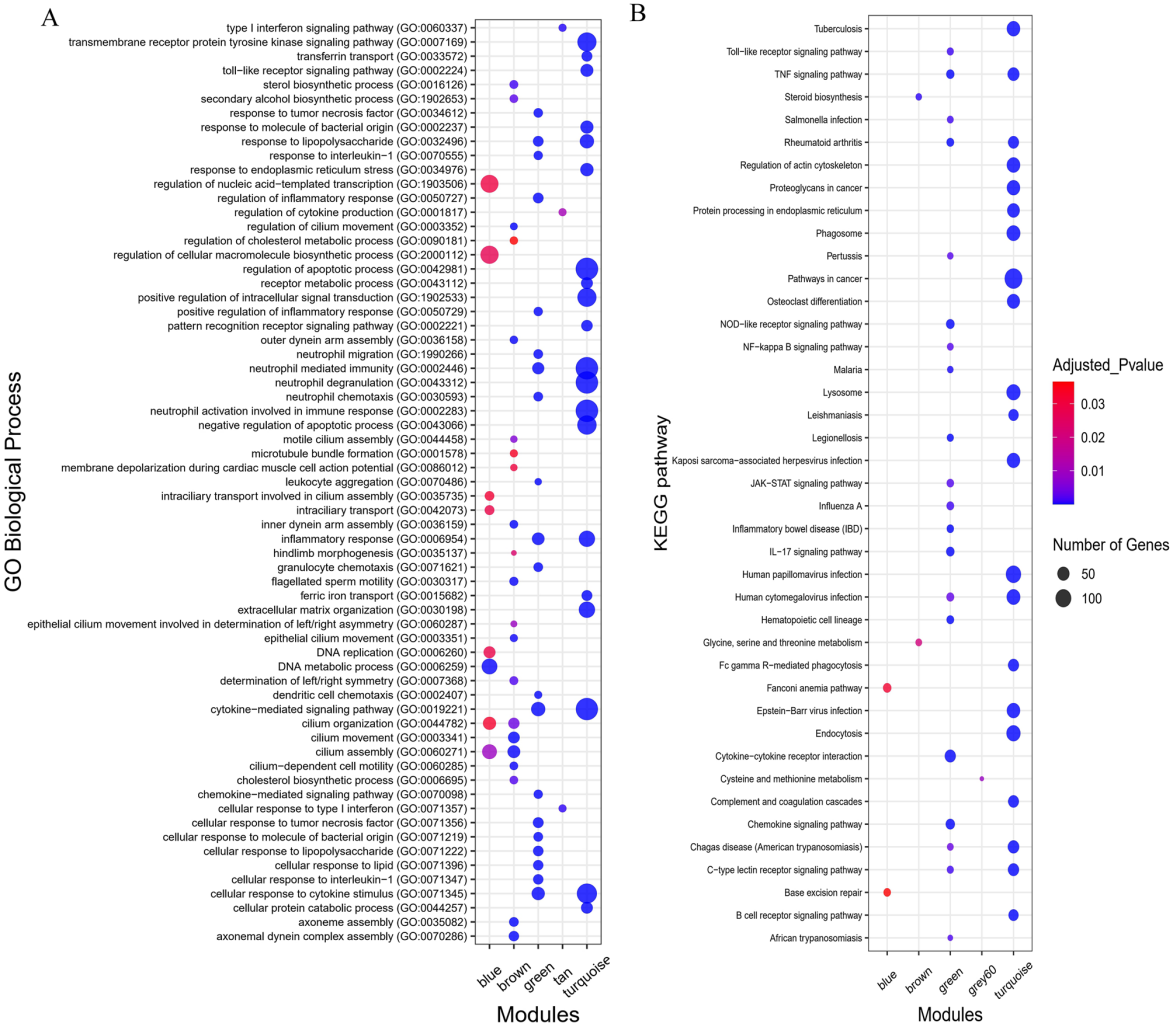
Functional enrichment analysis results (**A**) GO biological process (**B**) KEGG pathway of the non-preserved Rb-modules. Only the top 20 significant terms are displayed due to the large number of significant GO terms (biological process and KEGG pathway). The size of circles represents the enriched number of genes, and the color of circles represents the significance level of adjusted p-value. The blue color indicates the more significant terms within each module. As it can be seen, the turquoise module contains the highest number of significant terms based on GO biological process and KEGG pathway. Therefore, it can be considered one of the most important modules involved in the development of endometritis.

To assess the putative functions associated with the modules, all the identified Rb-modules and their hub genes were subjected to functional enrichment analysis, separately. Totally, 609 biological processes and 157 KEGG pathways were significantly enriched in six non-preserved modules including inflammatory response, response to lipopolysaccharide, regulation of MAP kinase activity, T cell chemotaxis, neutrophil migration cytokine-mediated signaling pathway, chemokine signaling pathway, IL-17 signaling pathway, TNF signaling pathway, NF-kappa B signaling and Toll-like receptor signaling pathway (Supplementary File S8, Fig. 5). The results suggesting that these genes might be related to endometritis development. Furthermore, the hub genes in the non-preserved Rb-modules found to be significantly enriched in 690 GO terms and 145 KEGG pathways. These hub genes were mostly enriched in the GO terms or KEGG pathways similar to results of the relevant modules (Supplementary File S9).

Enrichment analysis of the three semi-preserved Rb-modules showed that 10 biological processes and 11 KEGG pathways were significantly enriched. Sterol biosynthetic process, cholesterol metabolic process, regulation of alcohol biosynthetic process and regulation of steroid biosynthetic process were some of the GO terms. KEGG pathways were mainly associated with protein digestion and absorption, steroid biosynthesis, non-alcoholic fatty liver disease and ribosome biogenesis in eukaryotes (Supplementary File S10). Also, 29 GO terms and 12 KEGG pathways were significantly enriched in hub genes in semi-preserved modules which were according to the results of relevant modules (Supplementary File S11).

In three preserved modules, 118 biological processes and 14 KEGG pathways were significantly enriched. GO terms were related to translation, ncRNA processing, gene expression, ribosome biogenesis, mitotic cytokinesis, positive regulation of cell cycle process, regulation of cytokinesis, DNA recombination and DNA biosynthetic process (Supplementary File S12). KEGG pathways were mainly involved in cell cycle and ribosome. According to the results, preserved Rb-modules were enriched in the terms related to common activities of the cells and are less likely to be involved in endometritis development. Also, 170 GO terms and 12 KEGG pathways were significantly enriched in hub genes of the preserved Rb-modules and were similar to the results of the relevant modules (Supplementary File S13).

### Assigning the mb-modules to the Rb-modules and target prediction

To explore the potential molecular mechanisms responsible for endometritis, just non-preserved Rb-modules with significant functional enrichment results were more considered. In this way, MEs of mb-modules were observed to be correlated with MEs of Rb-modules using spearman correlation. Thereafter, mb-modules that were found to be negatively correlated with Rb-modules (adjusted p < 0.20 and correlation > 0.8), were considered as the potential regulators of those Rb-modules. Totally, six significant negative correlations were found among the four mb-modules and three Rb-modules (Table 1). Finally, in order to make a stronger biological connection between mb-modules and non-preserved Rb-modules, the target prediction was performed (Supplementary File S15). The summary of the target prediction results is presented in Table 2.

**Table 1:** Results of assignment of the mb-modules to the non-preserved Rb-modules.

mb-module (number of miRNAs)	Rb-module (number of genes)	Correlation	Adjusted p-value
Blue (60)	Blue (2368)	− 0.77	0.2
Blue (60)	Brown (1006)	− 0.84	0.2
Blue (60)	Turquoise (3188)	0.84	0.2
Brown (47)	Blue (2368)	− 0.75	0.2
Brown (47)	Brown (1006)	− 0.84	0.2
Brown (47)	Turquoise (3188)	0.78	0.2
Purple (15)	Blue (2368)	0.76	0.2
Purple (15)	Brown (1006)	0.94	0.2
Purple (15)	Turquoise (3188)	− 0.83	0.2
Yellow (31)	Brown (1006)	− 0.77	0.2

**Table 2:** The summary of the target prediction results for the mb-modules that were negatively correlated with mb-modules.

Software	mb-module (number of miRNAs)	Rb-module (number of genes)	Number of the unique predicted targets
miRanda	Blue (54)	Brown (1006)	525 mRNAs, 41 TFs and 8 lncRNAs
	Brown (44)	Brown (1006)	553 mRNAs, 35 TFs and 7 lncRNAs
	Purple (13)	Turquoise (3188)	997 mRNAs, 60 TFs and 28 lncRNAs
RNAhybrid	Blue (54)	Brown (1006)	412 mRNAs, 29 TFs and 7 lncRNAs
	Brown (44)	Brown (1006)	398 mRNAs, 30 TFs and 8 lncRNAs
	Purple (13)	Turquoise (3188)	326 mRNAs, 20 TFs and 16 lncRNAs
RNA22	Blue (54)	Brown (1006)	654 mRNAs, 45 TFs and 8 lncRNAs
	Brown (44)	Brown (1006)	645 mRNAs, 45 TFs and 8 lncRNAs
	Purple (13)	Turquoise (3188)	1122 mRNAs, 60 TFs and 32 lncRNAs
Common results of all softwares	Blue (54)	Brown (1006)	130 mRNAs, 15 TFs and 6 lncRNAs
	Brown (44)	Brown (1006)	142 mRNAs, 13 TFs and 2 lncRNAs
	Purple (13)	Turquoise (3188)	59 mRNAs, 4 TFs and 4 lncRNAs

### Detection of hub-hub genes and integrated regulatory network construction

**Figure 6:**
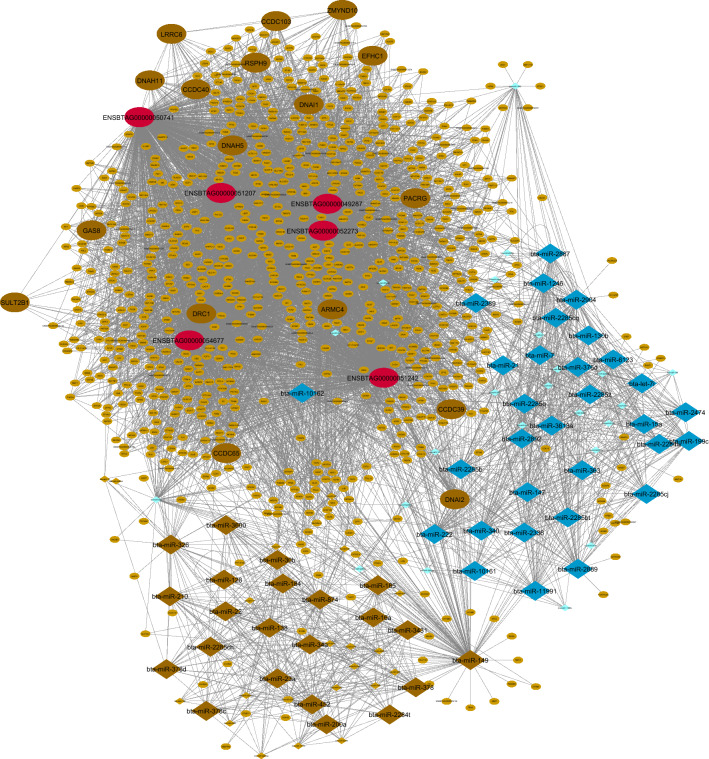
Integrated regulatory network of blue and brown mb-module and brown Rb-module. Small light brown circles represent hub genes, bold large red circles represent hub-hub lncRNAs and bold large brown circles represent hub-hub mRNAs in brown Rb-module. Also, small light blue and brown diamonds represent blue and brown mb-module and large bold diamonds represent hub miRNAs in these modules. Only the targets that have been predicted by all target prediction software are shown. Cytoscape software (version 3.7.2) (https://cytoscape.org/) was used to generate this Figure.

**Figure 7:**
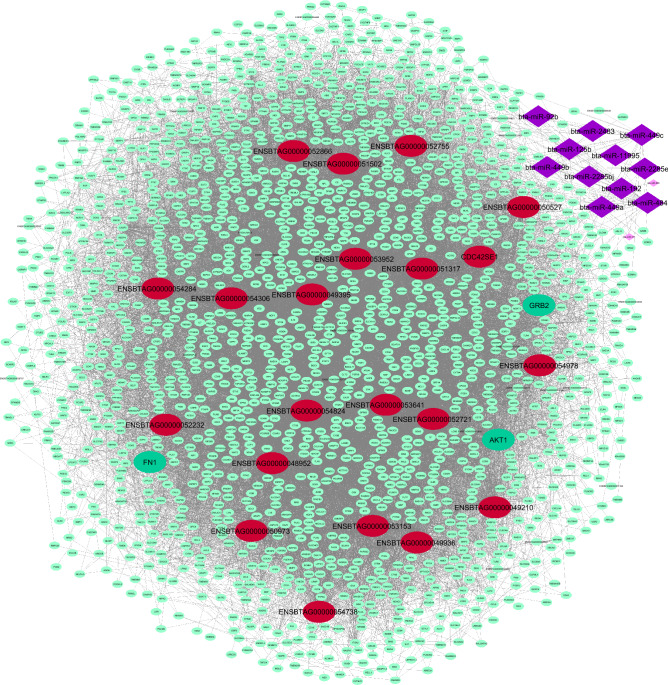
Integrated regulatory network of purple mb-module and turquoise Rb-module. Small light turquoise circles represent hub genes, bold large red circles represent hub-hub lncRNAs and bold large turquoise circles represent hub-hub mRNAs in turquoise Rb-module. Also, small light purple diamonds represent purple mb-module and large bold diamonds represent hub miRNAs in this module. Only the targets that have been predicted by all target prediction software are shown. Cytoscape software (version 3.7.2) (https://cytoscape.org/) was used to generate this Figure.

PPIs results of hub-mRNAs of the non-preserved Rb-modules, which were found to be negatively correlated with mb-modules, were obtained from the STRING database. Hence, two hub mRNA sets related to brown and turquoise Rb-modules were analyzed and all the obtained PPI-networks were significant (brown: 459 nodes, 1015 edges, and 1.0e−16 adjusted p-value; turquoise: 1639 nodes, 14,909 edges, and 1.0e−16 adjusted p-value). Subsequently, PPIs data were merged with the predicted interactions from WGCNA and then subjected to the hub–-hub genes’ identification. Totally, 47 hub–hub genes were identified in brown (containing 23 hub–hub genes, including 17 mRNAs and six lncRNAs) and turquoise (containing 24 hub-hub genes, including three mRNAs and 21 lncRNAs) Rb-modules. In order to construct an integrated regulatory network, the interactions obtained from STRING-PPIs, WGCNA-calculated co-expressed mRNAs, WGCNA-calculated co-expressed lncRNAs, and WGCNA-calculated co-expressed miRNAs were combined with the target prediction results. The summary of the integrated regulatory networks is presented in Table 3 (Supplementary File S16). The constructed integrated regulatory networks are displayed in Figs. 6 and 7.

**Table 3:** The summary of the interactions in the integrated regulatory networks.

Rb-module (total number of hub genes)	Brown (692 genes including 6 lncRNAs, 49 TFs + and 637 mRNAs)		Turquoise (1910 genes including 22 lncRNAs, 86 TFs and 1570 mRNAs)
Number of hub mRNAs that were analyzed in STRING	459		1639
Number of obtained interactions in STRING	1015		14,909
p-value of the network in STRING	1.0e−16		1.0e−16
Number of hub-mRNAs and hub-lncRNAs were imported into WGCNA	692		250
Number of interactions among genes and lncRNAs predicted by WGCNA	4131		4995
Number of hub-hub genes	23 (6 lncRNAs and 17 mRNAs)		24 (21 lncRNAs and 3 mRNAs)
Assigned mb-module to Rb-module (total number of miRNAs)	Brown (47)	Blue (60)	Purple (15)
Number of interactions among miRNAs predicted by WGCNA	Brown = 373	Blue = 641	PURPLE = 41
Number of assigned miRNAs to genes of Rb-modules in each mb-module	Brown = 22	Blue = 19	Purple = 7

## Discussion

Bovine endometritis is known as the most common uterine disease, which occurs following parturition and affects milk yield and reproductive performance and causes serious economic burden. Most of studies in this field, just consider differentially expressed genes without identifying relationships among genes and considering other biological factors including miRNAs and lncRNAs. Whereas assessing the interactions among all regulatory factors can provide a comprehensive insight into the mechanisms involved in endometritis pathogenesis. Herein, the integrated regulatory network approach was applied to help better understanding the functional networks/pathways contributing into the endometritis development. To do this, Rb-modules (including mRNAs, TFs, and lncRNAs) as well as mb-modules (including miRNAs) were constructed based on the WGCNA approach and then assigned to each other. Next, we focused on the non-preserved Rb-modules that were found to be negatively correlated with mb-modules. Negative correlations can be indicative of inverse interactions between Rb-modules and mb-modules. It is noteworthy that the loss of connectivity among genes in the non-preserved modules can be attributed to the abnormal expression of some genes under the endometritis condition, which likely are key factors. In order to explore these potential genes that may be related to the occurrence and development of endometritis, different methods were applied, including assigning mb-modules to Rb-module, functional enrichment analysis, PPI network construction, and hub and hub-hub genes identification along with considering the relevant literature review. In addition, the target prediction analysis was used to assess the assigned genes (from Rb-modules) to miRNAs (from mb-modules) at the sequence level. Our findings provided evidence that the dysregulated genes may be associated with the development of endometritis. In this study, two most important non-preserved Rb-modules were found, namely turquoise and brown modules, which not only were negatively connected to the mb-modules, but also their hub genes were significantly connected in terms of PPI network. Therefore, genes with a high degree of connectivity within these modules, are also linked at the protein–protein interaction level. Moreover, the results of the functional enrichment analysis of these modules showed that some terms were associated with most of the features of endometritis and some of them were only related to the promotion of the establishment of the endometritis.

**Figure 8:**
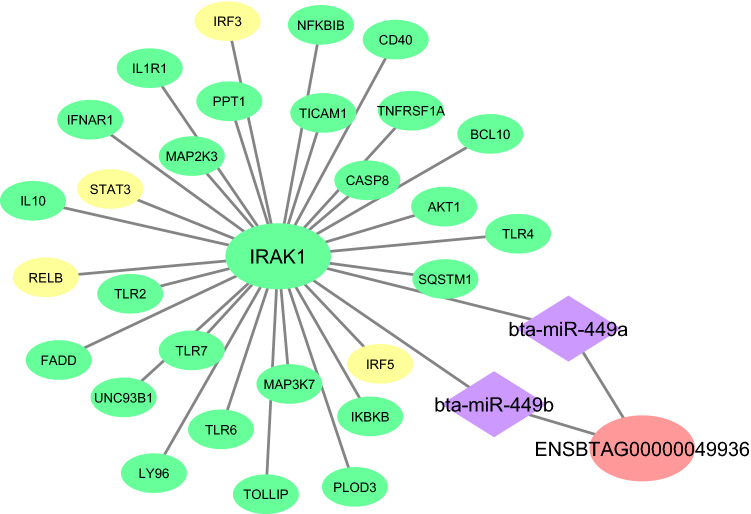
The constructed integrated regulatory sub-network related to *IRAK1* gene in turquoise Rb-module. The green and yellow circles indicate hub genes and hub TFs related to *IRAK1*, respectively. The purple diamonds represent the hub miRNAs that be predicted to regulate *IRAK1* and hub-hub lncRNA *ENSBTAG00000049936*. Cytoscape software (version 3.7.2) (https://cytoscape.org/) was used to generate this Figure.

The functional enrichment analysis of the turquoise Rb-module revealed some enriched terms related to both inflammation and infection, including cytokine-mediated signaling pathway, Toll-like receptor signaling pathway, inflammatory response, positive regulation of NF-kappaB transcription factor activity, and MAPK cascade. Furthermore, the results of KEGG pathway analysis indicated that TNF signaling pathway, B cell receptor signaling pathway, NF-kappa B signaling pathway, Toll-like receptor signaling pathway, and MAPK signaling pathway are significantly enriched in this module. Besides, the bacterial infection of the endometrium was found to induce an inflammatory response by the secretion of chemokines and cytokines^57^. Of note, Toll-like receptors (TLRs) are the crucial receptors on endometrial cells and macrophages, which are used for recognizing pathogen-associated molecular patterns (PAMPs) present on bacterial cell walls like lipopolysaccharide (LPS)^58^. After recognizing PAMPs by TLRs, Myeloid differentiation factor 88 (*MyD88*) interacted with *IL-1* receptor-associated kinase-4 (*IRAK-4*) and formed the *MyD88-IRAK-4* complex. Subsequently, *IRAK-1* and *IRAK-2* were recruited, which can lead to the phosphorylation of IRAKs. Correspondingly, the phosphorylation of these kinases was also found to have the ability of leading to the recruitment of other proteins. Finally, the nuclear factor-kB (*NF-kB*) signaling and mitogen-activated protein kinase (MAPK) signaling pathways were activated. The activation of these downstream signaling pathways led to a cascade of inflammatory response as well as the secretion of chemokines and cytokines^58^. Interestingly, in our study, *IRAK1* was detected as the hub gene in turquoise module, which is known as one of the most important genes in TLRs signaling pathway. In the current study, intersection of the results of the three target prediction software revealed that *IRAK1* is a potential target of hub miRNAs, including *bta-miR-449a* and *bta-miR-449b*. Accordingly, these two miRNAs could also target hub-hub lncRNA *ENSBTAG00000049936* in turquoise module. It was confirmed that *miR-449a* is up-regulated in children’s with inflammatory bowel disease (IBD) and plays some roles in inflammatory response. The inhibition of *miR-449a* has been previously shown to inhibit inflammatory response^59^. Jianga et al. in their study have reported that *miR-449a* plays a crucial role in patients with atherosclerosis via regulating *TNF-a* expression^60^. Moreover, the results of the study by CAI et al. demonstrated that *IL-6* is targeted by *miR-449b-3p*, which consequently affects the JAK2/STAT3 signaling pathway^61^. The constructed integrated regulatory sub-network related to *IRAK1* in turquoise Rb-module is shown in Fig. 8.

**Figure 9:**
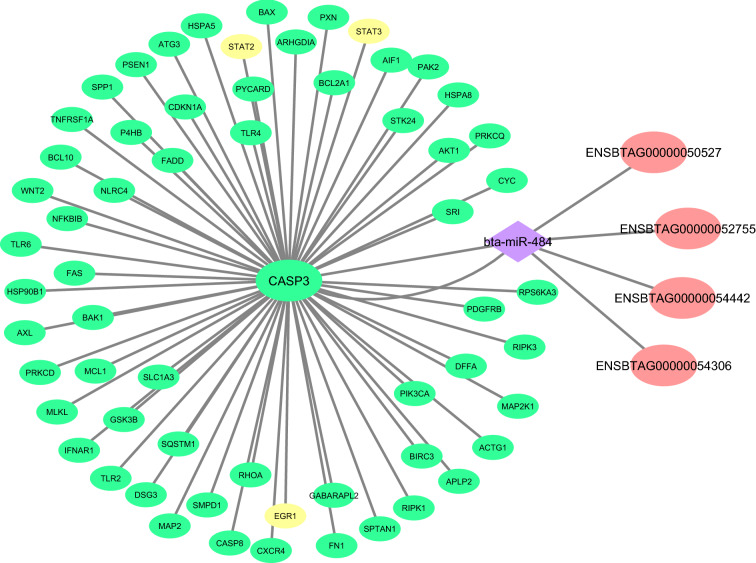
The constructed integrated regulatory sub-network related to *CASP3* in turquoise Rb-module. The green and yellow circles indicate hub genes and hub TFs related to casp3, respectively. The purple diamond represents hub miRNA that be predicted to regulate *CASP3* and hub-hub lncRNAs including *ENSBTAG00000050527*, *ENSBTAG00000052755* and *ENSBTAG00000054306*. Cytoscape software (version 3.7.2) (https://cytoscape.org/) was used to generate this Figure.

Among the genes in turquoise module, *CASP3* (which is a hub gene) was previously found to be associated with inflammatory response and predicted to be targeted by hub *bta-miR-484* (based on both the miRanda and RNAhybrid results). Of note, several hub-hub lncRNAs in turquoise module, including *ENSBTAG00000050527*, *ENSBTAG00000052755*, and *ENSBTAG00000054306*, are also targeted by *bta-miR-484*. In mammals, caspase gene family consists of 15 members that can be grouping into inflammatory caspases and apoptotic caspases^62^. In this regard, apoptotic caspases have an association with cellular dismantling, while inflammatory caspases only mediate the activation of inflammatory cytokines^62^. Caspase-3 (*CASP3*) is known as a key mediator of apoptosis^62^. In addition, it has been demonstrated that *CASP3* plays a crucial role in the differentiation of various cells such as monocytes, osteoblasts, and platelet^63^. The caspase-3 family, including caspases-3 and -7, plays an important role in activating pro-inflammatory cytokines^64^. On the other hand, myocardial cells can prevent ischemia–reperfusion injury via the role of *mir-484* in suppressing both caspase-3 and caspase-9 expressions during cardiomyocyte apoptosis. Besides, *mir-484* reduces the expressions of *IL-6*, *TNF-α*, and *IL-1β*^65^. The constructed integrated regulatory sub-network regarding *CASP3* in turquoise Rb-module is shown in Fig. 9.

Some of the highly connected genes in turquoise module, including *AKT1* (hub-hub), *STAT3* (hub), *IL10* (hub), *TLR4* (hub), *CD68* (hub), and *FN1* (hub-hub), have been reported to be related to immune response and inflammation. The potential regulatory networks of these genes are available in Supplementary File S17. *AKT1* is a primary effector molecule of PI3K-AKT signaling pathway, acting as a suppressor for the *NF-κB* activation^66^. It was previously found that *miR-29a* could regulate the *NF-κB* pathway via targeting *AKT1*, thereby promoting inflammatory responses^67^. In contrast, Kane et al. in their study have reported that *AKT1* plays roles in the IκB proteins degradation and subsequently in the *NF-κB* activation^68^. In the patients with endometrial cancer, some hub genes, including *PBK*, *BIRC5*, *AURKA*, *GTSE1*, *KNSTRN*, and *PSMB10*, were detected to be associated with *AKT1*. Hence, the higher expression of *AKT1* was observed to be significantly associated with developing the endometrial cancer^69^. *STAT3*, as one of the hub TFs in turquoise Rb-module, is known as a regulator of expression of many genes, inducing some important cytokines. Furthermore, it is considered as a mediator of both innate and adaptive immunities. Notably, lack of *STAT3* in immune cells induces severe inflammation in response to pathogens^70^. In the bovine endometrium, *IL-6* acts as an activator of *STAT3*, the activation of which more increases in the secretion of both *IL-6* and *IL-8*^71^. It has been shown that phosphorylation and activation of *STAT3* in the endometrium are essential for a successful implantation^72^. Pro-inflammatory cytokines could initiate and increase the inflammatory response, while anti-inflammatory cytokines only modulate the pro-inflammatory cytokines^73^. *IL-10* is known as one of the anti-inflammatory cytokines, deficiencies in which can lead to tissue damage^74^. As mentioned earlier, TLRs, which are involved in inflammation and immune system, play some crucial roles in initiating inflammatory response. TLRs could also activate immune cells via two separate signaling pathways, namely *MYD88* and *TRIF*. Furthermore, *TLR4* uses both signaling pathways for the activation of immune cells. Several investigations have reported *TLR4* as the main molecule in the development of the pro-inflammatory-based diseases^75^. Several studies have previously shown the upregulation of *CD68* expression in macrophages in response to inflammatory stimuli. Accordingly, it is also commonly used as a histochemical/cytochemical marker of inflammation^76^. Fibronectin (*FN1*), which is an extracellular matrix molecule, has been identified as an activator of TLRs^77^. The interaction between FN and integrin β1 in macrophages results in reinforcing Toll-like receptor 2/4 (*TLR2*/*TLR4*) signaling pathways as well as phagocytosis by macrophages^78^. Generally, due to the functional roles of the above-mentioned genes in inflammation and immune system, these hub genes as well as the suggested integrated regulatory network may be involved in both the pathogenesis and progression of endometritis. As well, their possible cellular roles and the biological mechanisms related to this infection can be further assessed.

In the current study, cilium organization and cilium movement GO terms were significantly enriched in brown Rb-module. In this regard, Samatha et al. in their study investigated endometrial biopsies samples obtained from infertile buffaloes in terms of the histopathological and immunohistochemical properties and as a result, they reported that surface epithelial cells contain few ciliated and non-ciliated cells. Moreover, they observed the loss of both cilia and microvilli of the surface epithelium in acute endometritis cases^79^. Cilia are microtubule-based, conserved organelles growing from basal bodies^80^. It has been hypothesized that cilia play the role of antennae by the signal detection^80^. In this context, the role of primary cilia in NF-κB signaling pathway through the regulation of *IKK* activity has been demonstrated, as well^81,82^. Additionally, Beak et al. in their study reported a link between the expression of pro-inflammatory cytokines and ciliary function^83^. The disruption of ciliary intraflagellar transport (IFT) could alter the cell response to *IL-1β*, which supports the existence of a putative link between cilia and inflammation^84^. IFT includes motor proteins, kinesins, and dyneins and plays a crucial role in transporting ciliary proteins within the cilium^85^. Some of the highly connected genes related to cilium organization in brown Rb-module are *CCDC39*, *CCDC40*, *ZMYND10*, *DNAAF11* and *FOXJ1*.

**Figure 10:**
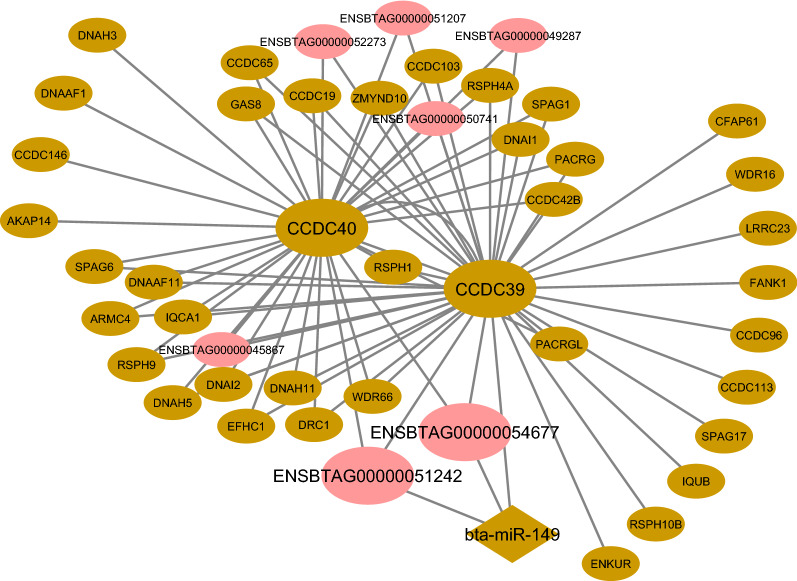
The constructed integrated regulatory sub-network related to *CCDC40* and *CCDC39* in brown Rb-module. The pink circles indicate hub and hub-hub lncRNAs. The brown diamond represents hub miRNA that be predicted to regulate *CCDC39* and hub-hub target lncRNAs including *ENSBTAG00000051242* and *ENSBTAG00000054677*. Cytoscape software (version 3.7.2) (https://cytoscape.org/) was used to generate this Figure.

*CCDC40* is one of the identified hub–hub genes in the brown Rb-module, which is required for axonemal assembly and the proper formation and/or maintenance of cilia. In addition, it is essential for both cytoplasmic pre­assembly and transportation of the axonemal components, including *CCDC39*, *GAS11*, and *DNALI1*^86^. The key roles of *CCDC39* and *CCDC40*, which are involved in axonemal disorganization and inner dynein arm loss, in patients with primary ciliary dyskinesia (PCD) have been demonstrated previously. These genes are known as major candidates for genetic testing in families affected by this ciliary phenotype^87^. According to the target prediction results of miRanda, RNAhybrid, and RNA22, *CCDC39* was found as a potential target of *bta-miR-149* hub miRNA. Moreover, hub-hub lncRNAs, including *ENSBTAG00000051242* and *ENSBTAG00000054677*, were observed to be targeted by *bta-miR-149*. It is worth noting that the regulatory role of *miR-149* in inflammation has been reported in several previous studies^88,89^. The constructed integrated regulatory sub-network regarding both *CCDC40* and *CCDC39* in brown Rb-module is displayed in Fig. 10.

**Figure 11:**
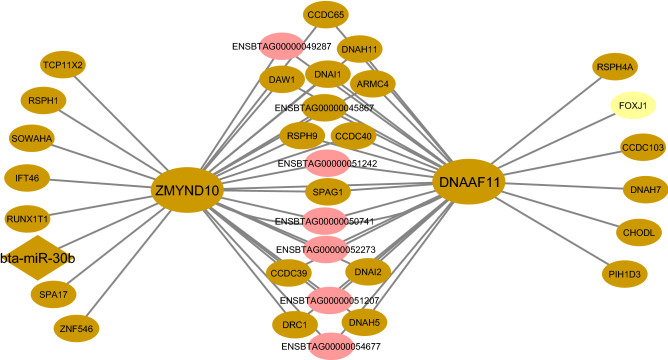
The constructed integrated regulatory sub-network related to *ZMYND10* and *DNAAF11* in brown Rb-module. The pink circles indicate hub-hub lncRNAs related to the mentioned genes. The brown diamond represents hub miRNA that be predicted to regulate *ZMYND10*. The yellow circle indicates hub TF. Cytoscape software (version 3.7.2) (https://cytoscape.org/) was used to generate this Figure.

In the present study, *ZMYND10* was identified as a hub-hub gene potentially predicted to be targeted by hub miRNA *bta-miR-30b*. In this regard, *ZMYND10* plays an important role in the dynein-arm synthesis, assembly or transport. In flies, *ZMYND10* is primarily considered as a cytoplasmic component of cells containing motile cilia^90^. Moreover, both *ZMYND10* and *DNAAF11* are assembled into a protein complex, which plays a role in the transcriptional regulation of some dynein components^91^. Moreover, the regulatory role of *miR-30b* has been reported in inflammation^92^. The constructed integrated regulatory sub-network regarding both *ZMYND10* and *DNAAF11* in brown Rb-module is provided in Fig. 11.

**Figure 12:**
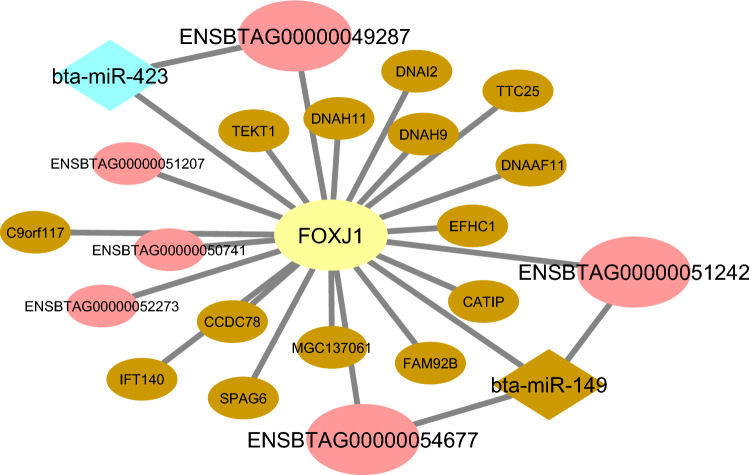
The constructed integrated regulatory sub-network related to *FOXJ1* in brown Rb-module. The pink circles indicate hub-hub lncRNAs related to the mentioned gene. The brown and blue diamond represents miRNAs that be predicted to regulate *FOXJ1*. Cytoscape software (version 3.7.2) (https://cytoscape.org/) was used to generate this Figure.

One of the hub TFs in brown Rb-module is *FOXJ1*, which is involved in the transcriptional regulation of those genes that encode essential components for the synthesis and function of motile cilia^93^. According to the results of the target prediction analysis, *FOXJ1* is targeted by *bta-miR-423* in blue mb-module and *bta-miR-149* (identified as a hub miRNA) in brown mb-module. Accordingly, these miRNAs could also target hub-hub lncRNAs, including *ENSBTAG00000049287*, *ENSBTAG00000051242*, and *ENSBTAG00000054677* in the brown Rb-module. Interestingly, the roles of both miRNAs (*miR-149* and *miR-423*) in inflammatory response have been reported, reinforcing the potential regulatory roles of these genes in endometritis^89,94^. The constructed integrated regulatory sub-network regarding *FOXJ1* in brown Rb-module is provided in Fig. 12.

In summary, our results suggest, in accordance with literature, the potential role of the brown Rb-module in ciliogenesis and inflammation, which may provide a foundation for obtaining further understanding on the underlying molecular mechanisms of endometritis development in the bovine. In addition, our findings showed that the integration of mRNAs, lncRNAs, and miRNAs based on the WGCNA networks along with different data such as PPI and miRNA target prediction results can be considered as a robust approach to provide greater insights on the disease‐related biological process.

## Conclusion

In the present study, integrated regulatory networks related to bovine endometritis were constructed by assigning the mb-modules to the non-preserved Rb-modules. These modules were constructed based on the gene co-expression approach (WGCNA) and were also assessed by PPI data, functional enrichment analysis and target prediction analysis. Moreover, only highly connected genes (hubs and hub-hubs) were considered, which are the key components of the networks and more likely play an important role in endometritis. Two important non-preserved Rb-modules (brown and turquoise) were found that were assigned to three mb-modules (brown, blue and purple). In addition, several sub-networks in brown and turquoise modules were identified, including mRNAs (such as *IRAK1*, *AKT1*, *STAT3*, *CCDC39* and *ZMYND10*), lncRNAs (such as *ENSBTAG00000049936*, *ENSBTAG00000050527*, *ENSBTAG00000054306*, *ENSBTAG00000051242* and *ENSBTAG00000049287*) as well as miRNAs (such as *bta-miR-449a*, *bta-miR-484*, *bta-miR-30b*, *bta-miR-149* and *bta-miR-423*), as potential pathways/genes that may contribute to the progression of bovine endometritis and could be regarded as biomarkers and therapeutic targets of this infection. The proposed integrated regulatory networks can provide a basis for additional inspection of the regulatory mechanisms of endometritis; however, further experimental works are needed to validate our findings and elucidate the importance of these networks in bovine endometritis.

## Supplementary Information

Supplementary File S1.

Supplementary File S2.

Supplementary File S3.

Supplementary File S4.

Supplementary File S5.

Supplementary File S6.

Supplementary File S7.

Supplementary File S8.

Supplementary File S9.

Supplementary File S10.

Supplementary File S11.

Supplementary File S12.

Supplementary File S13.

Supplementary File S14.

Supplementary File S15.

Supplementary File S16.

Supplementary File S17.
