# Supplementary material for: An integrated analysis of mRNAs, lncRNAs, and miRNAs based on weighted gene co-expression network analysis involved in bovine endometritis
Source: Sci Rep. 2021 Sep 10;11:18050. doi: 10.1038/s41598-021-97319-y (PMC8433134; doi:10.1038/s41598-021-97319-y)
Supplement: Supplementary file 14 — Supplementary File S14. [file 41598_2021_97319_MOESM14_ESM.docx]

**An Integrated Analysis of mRNAs, lncRNAs, and miRNAs Based on Weighted Gene Co-Expression Network Analysis Involved in Bovine Endometritis**

**Negin Sheybani, Mohammad Reza Bakhtiarizadeh*, Abdolreza Salehi**

Department of Animal and Poultry Science, College of Aburaihan, University of Tehran, Tehran, Iran.

* **Correspondence:**

Mohammad Reza Bakhtiarizadeh

[mrbakhtiari@ut.ac.ir](mailto:mrbakhtiari@ut.ac.ir), ORCID ID: ​0000-0001-5336-6987

**Supplementary File S14:** Correlation between the MEs of Rb-modules and mb-modules
